# MECP2 Increases the Pro-Inflammatory Response of Microglial Cells and Phosphorylation at Serine 423 Regulates Neuronal Gene Expression upon Neuroinflammation

**DOI:** 10.3390/cells10040860

**Published:** 2021-04-09

**Authors:** Rebekka Wittrahm, Mari Takalo, Mikael Marttinen, Teemu Kuulasmaa, Petra Mäkinen, Susanna Kemppainen, Henna Martiskainen, Tuomas Rauramaa, Ian Pike, Ville Leinonen, Teemu Natunen, Annakaisa Haapasalo, Mikko Hiltunen

**Affiliations:** 1 Institute of Biomedicine, Yliopistonranta 1E, University of Eastern Finland, 70211 Kuopio, Finland; rebekka.wittrahm@uef.fi (R.W.); mari.takalo@uef.fi (M.T.); mikael.marttinen@uef.fi (M.M.); teemu.kuulasmaa@uef.fi (T.K.); petra.makinen@uef.fi (P.M.); susanna.kemppainen@uef.fi (S.K.); henna.martiskainen@uef.fi (H.M.); teemu.natunen@uef.fi (T.N.); 2 Structural and Computational Biology Unit, European Molecular Biology Laboratory, Meyerhofstraße 1, 69117 Heidelberg, Germany; 3 Department of Pathology, Kuopio University Hospital, 70029 Kuopio, Finland; tuomas.rauramaa@kuh.fi; 4 Unit of Pathology, Institute of Clinical Medicine, University of Eastern Finland, 70210 Kuopio, Finland; 5 Proteome Sciences plc, Hamilton House, London WC1H 9BB, UK; ian.pike@proteomics.com; 6 Department of Neurosurgery, Kuopio University Hospital, 70029 Kuopio, Finland; ville.leinonen@kuh.fi; 7 Unit of Neurosurgery, Institute of Clinical Medicine, University of Eastern Finland, 70211 Kuopio, Finland; 8 A.I. Virtanen Institute for Molecular Sciences, Neulaniementie 2, 70211 Kuopio, Finland; annakaisa.haapasalo@uef.fi

**Keywords:** Alzheimer’s disease, MECP2, microglia, neuroinflammation, post-translational modifications, synaptic markers

## Abstract

Methyl-CpG-binding protein 2 (MECP2) is a critical transcriptional regulator for synaptic function. Dysfunction of synapses, as well as microglia-mediated neuroinflammation, represent the earliest pathological events in Alzheimer’s disease (AD). Here, expression, protein levels, and activity-related phosphorylation changes of MECP2 were analyzed in post-mortem human temporal cortex. The effects of wild type and phosphorylation-deficient MECP2 variants at serine 423 (S423) or S80 on microglial and neuronal function were assessed utilizing BV2 microglial monocultures and co-cultures with mouse cortical neurons under inflammatory stress conditions. MECP2 phosphorylation at the functionally relevant S423 site nominally decreased in the early stages of AD-related neurofibrillary pathology in the human temporal cortex. Overexpression of wild type MECP2 enhanced the pro-inflammatory response in BV2 cells upon treatment with lipopolysaccharide (LPS) and interferon-γ (IFNγ) and decreased BV2 cell phagocytic activity. The expression of the phosphorylation-deficient MECP2-S423A variant, but not S80A, further increased the pro-inflammatory response of BV2 cells. In neurons co-cultured with BV2 cells, the MECP2-S423A variant increased the expression of several genes, which are important for the maintenance and protection of neurons and synapses upon inflammatory stress. Collectively, functional analyses in different cellular models suggest that MECP2 may influence the inflammatory response in microglia independently of S423 and S80 phosphorylation, while the S423 phosphorylation might play a role in the activation of neuronal gene expression, which conveys neuroprotection under neuroinflammation-related stress.

## 1. Introduction

Alzheimer’s disease (AD) is the most common form of dementia and one of the leading causes of cognitive disability worldwide. The key pathological features of AD include β-amyloid plaques, consisting of toxic forms of amyloid-β (Aβ) peptides, and neurofibrillary tangles, composed of hyperphosphorylated tau protein [1]. Based on genetic studies, as well as large-scale transcriptomics and proteomics screenings, microglial activation plays a major role in AD pathogenesis [2]. Microglia are the major cell type responsible for inflammatory responses, as well as phagocytosis of toxic Aβ aggregates and apoptotic neurons in the brain. Conversely, prolonged activation of microglia creates a toxic inflammatory environment, which can be detrimental to synapses and neurons [3]. Synaptic dysfunction is the earliest pathological feature of AD and underlies cognitive decline in the early stages of the disease [1].

Methyl-CpG-binding protein 2 (MECP2) is a multifunctional protein that can modulate gene expression by binding to methylated cytosines and by interacting with chromatin modifiers [4]. MECP2 is one of the key regulators of synaptic plasticity and has been suggested to play a role in microglial activation [5,6]. Although abnormal MECP2 function is known to disrupt synaptic plasticity and neuronal function in various neurological diseases [5,7,8,9], there are only a few studies describing its potential link to AD [10,11]. In human post-mortem hippocampal tissue, the levels of MECP2 protein were significantly elevated in AD as compared to controls [12]. Recently, MECP2 was suggested to regulate tau phosphorylation and deposition, implicating a direct association to AD pathology [11]. Upregulation of the MECP2-e2 isoform (corresponding to human MECP2 isoform 1) was found to enhance Aβ-induced apoptosis in cultured primary mouse neurons [13]. Functional versatility of MECP2, including its affinity for different binding partners, transcriptional regulation of specific gene sets, and effects on neuronal plasticity, is modulated by post-translational modifications (PTM), such as phosphorylation [9,10,14]. Thus, controlling MECP2 levels, activity, or downstream targets by targeting MECP2 PTMs could be a feasible approach for developing specific therapies against the detrimental effects of MECP2 during AD pathogenesis.

Phosphorylation of mouse MECP2 at serine 421 (corresponding to S423 in human MECP2 isoform 1) upon neuronal activity has been shown to facilitate the release of a transcription factor from the MECP2 transcriptional repressor complex, coinciding with increased brain-derived neurotrophic factor (BDNF) expression, enhanced synaptic function, and improvement of learning and memory, but the role of these processes in the context of AD is not known [15]. Notably, BDNF is a major modulator of synaptic density and plasticity, and one of the main targets of regulation through MECP2 [16]. Phosphorylation of MECP2 at serine 80 (S80) is the most abundant in neurons under resting conditions, whereas neuronal activity induces MECP2 dephosphorylation at S80. In addition, phosphorylation of S80 has been shown to facilitate binding of MECP2 to chromatin [17]. Altogether, these findings suggest that phosphorylation might play a key role in regulating MECP2 levels and functionality, and thus in the modulation of key pathological features of AD.

In recent years, the role of glial cells has been recognized in conditions with MECP2 deficiency [18,19,20]. The expression of wild type MECP2 in myeloid cells has been shown to arrest Rett syndrome disease pathology in *Mecp2*-null mice [21]. Phagocytic activity of microglia was found to play a crucial role in this process but the results could not be replicated in a later study [22]. Conditioned medium from *Mecp2*-null microglia negatively altered dendritic morphology of cultured hippocampal neurons through elevating the levels of glutamate [20]. Later, MECP2 has been found to act as a transcriptional repressor of the major glutamine transporter *SNAT1* (also known as *SLC38A1*), leading to glutamate overproduction in *Mecp2*-null microglia [9]. The role of microglial MECP2 has almost exclusively been studied in *Mecp2*-knockout mouse models but the impact of MECP2 PTMs has not yet been characterized in microglia or in the context of neuroinflammatory processes associated with AD. Thus, co-cultures of mouse primary neurons and primary microglia, or BV2 secondary microglial cells treated with lipopolysaccharide (LPS) and interferon-γ (IFNγ) are a useful tool to model certain aspects of AD-associated neuroinflammation in vitro [23]. Despite the fact that this model does not recapitulate the chronic inflammation associated with AD, LPS has been shown to induce similar microglia-mediated synaptic failure as Aβ and epigenetically suppress the synaptic expression of specific genes involved in learning and memory in vivo [10,24]. Recently, it has been suggested that LPS induces cognitive impairment and neuroinflammation via activation of the NF-kB signaling pathway in microglia [25]. In turn, NF-kB has been shown to be a key mediator of inflammation in AD brain tissue [26]. Furthermore, a comparison of primary microglia and BV2 cells in neuron-microglia co-cultures under LPS and IFNγ-induced neuroinflammation indicated similar outcomes in terms of neurotoxicity, inflammatory response, and the generation of nitric oxide (NO), which results from LPS-induced NF-kB activation [23,25,26].

Most of the studies addressing phosphorylation sites of MECP2 have focused on neurons. To expand this view beyond neurons, we have here investigated the effects of elevated MECP2 isoform 1 levels in BV2 microglial cells upon LPS and IFNγ-induced inflammation. We found that overexpression of wild type MECP2 (MECP2-WT) led to an increased expression of pro-inflammatory cytokines and decreased phagocytic activity of BV2 cells. Furthermore, phospho-proteomics data obtained from human post-mortem brain samples revealed a decrease in MECP2 S423 phosphorylation in the early stages of AD with respect to AD-related neurofibrillary pathology. The expression of S80A and S423A phosphorylation-deficient variants of MECP2 did not lead to major differences in the mRNA levels of pro-inflammatory cytokines or phagocytic activity of the BV2 cells compared to MECP2-WT expression. In contrast, the expression of the MECP2 S423A variant in mouse primary cortical neurons co-cultured with BV2 microglial cells upon induction of inflammation led to expressional changes of several different transcripts that are important for neuronal and synaptic maintenance and protection, including *Bdnf* and *Mapt*, encoding tau protein. Collectively, our findings from neuron-BV2 co-cultures underline the important regulatory role of the S423 MECP2 phosphorylation site under neuroinflammation-related neuronal stress.

## 2. Materials and Methods

### 2.1. Transcptomic, Proteomic, and Phospho-Peptide Analysis of Human Brain Samples

Global transcriptomics, proteomics, and phospho-peptide as well as soluble Aβ42 analyses were available from neuropathologically validated post-mortem temporal cortical samples, as described in detail previously [27]. Similarly, Aβ42, phosphorylated tau (p-tau), and total-tau measurements from cerebrospinal fluid (CSF) samples were available from a subset of individuals in the post-mortem cohort, as described previously [28].

### 2.2. BV2 Cell Culture

Immortalized murine microglial BV2 cells [29] were cultured in RPMI-1640 medium (R0883, Sigma–Aldrich, St. Louis, MO, USA) supplemented with 10% (*v*/*v*) fetal bovine serum (FBS, 10270-106, Gibco, Waltham, MA, USA), 2 mM L-glutamine (17-605E, Lonza, Basel, Switzerland), and 1 U/mL penicillin-streptomycin (DE17-602E, Lonza, Basel, Switzerland) in 15-cm cell culture dishes (150468, Nunc A/S, Roskilde, Denmark). Cells were maintained at 37 °C and 5% CO_2_ and sub-cultured at a split ratio of 1:5 by gently scraping the cells into fresh culture medium when they reached a confluence of 80–90%.

### 2.3. Co-Culture of Mouse Primary Cortical Neurons and BV2 Cells

Mouse primary cortical neurons were collected from JAXC57BL/6J mice on embryonic day 18, as described previously [30]. In brief, cortices were dissected, and a single-cell suspension was prepared by trypsin digestion followed by trituration. Cells were plated in Neurobasal medium (12348017, Gibco, Waltham, MA, USA) supplemented with 1× B-27 (17504044, Gibco, Waltham, MA, USA), 2 mM L-glutamine and 1% (*v*/*v*) penicillin-streptomycin (feeding medium). Plating was done on poly-D-Lysine (P6407, Sigma–Aldrich, St. Louis, MO, USA) -coated 48-well or 24-well plates (150687 or 142475, Nunc A/S, Roskilde, Denmark) at a density of 2 × 10^5^ cells/well or 4 × 10^5^ cells/well, respectively. After transduction of the neurons (described below), co-cultures were prepared as outlined before [23]. Briefly, on day 6 in vitro (DIV6), BV2 cells suspended in Neurobasal medium were added to the neurons at 1:5 ratio (BV2:neurons) and let to settle down for two hours before starting further treatments.

### 2.4. Lentiviral Vectors

Lenti open reading frame (ORF) clones of pLenti-CMV-C-Myc-DDK (Control) and human pLenti-CMV-MECP2-C-Myc-DDK (WT) were obtained from OriGene (PS100064, RC202382L1, OriGene Technologies, Rockville, MD, USA). MECP2-Myc-DDK was subjected to site-directed mutagenesis (QuikChange Lightning Multi Site-Directed Mutagenesis Kit, 210515, Agilent Technologies, Santa Clara, CA, USA) to create MECP2-S80A-Myc-DDK and MECP2-S423A-Myc-DDK constructs. Briefly, pLenti-CMV-MECP2-C-Myc-DDK plasmid DNA was amplified with a single mutagenic primer (5′-GGAAGCTTCTGCCGCCCCCAAACAGCG-3′ or 5′-CCCAGAGGAGGCGCACTGGAGAGCG-3′). The original plasmid was then digested with DpnI restriction enzyme and the mutagenized plasmid was directly transformed into One Shot TOP10 Chemically Competent *E. coli* (C404010, Invitrogen, Carlsbad, CA, USA). Sanger sequencing was used to confirm the introduction of the point mutation.

All three MECP2 variants, including the Myc tag, were subcloned into the bicistronic pLVX-EF1α-IRES-ZsGreen1 lentiviral expression vector (632187, Takara, Mountain View, CA, USA) with the In-Fusion HD Cloning Plus Kit (638909, Takara, Mountain View, CA, USA) following the manufacturer’s instructions. In brief, MECP2-Myc coding sequence (CDS) was amplified using primers with a template-specific and a pLVX-specific portion (5′-GAGCGGCCGCGGATCCATGGTAGCTGGGATGTTAGGG-3′ and 5′-GAGAGGGGCGGGATCCTTACAGATCCTCTTCTGAGATGAG-3′). PCR products were purified with NucleoSpin PCR Clean-Up Kit (740658.1, Takara, Mountain View, CA, USA) and together with linearized pLVX plasmid DNA added to the InFusion cloning reaction premix. After incubation for 15 min at 50 °C, plasmid DNA was transformed into Stellar Competent Cells (636767, Takara, Mountain View, CA, USA). Integrity of the insert sequence was confirmed through Sanger sequencing. All plasmids were packed into third-generation self-inactivating lentiviral particles by the National Virus Vector Laboratory (University of Eastern Finland, Kuopio, Finland).

### 2.5. Lentivirus Mediated Transduction and Treatment of Cells

For transduction with monocistronic lentiviral particles, BV2 cells were plated on 12-well cell culture plates (150628, Nunc A/S, Roskilde, Denmark) at a density of 2 × 10^5^ cells/well in 1 mL culture medium 2 h prior to transduction. Lentiviral particles were added to each well in 110 µL phosphate-buffered saline (PBS) at a multiplicity of infection (MOI) of 20. After 24 h of lentiviral infection, the cells were washed twice with culture medium to remove residual lentiviral particles. Cells were treated immediately with 200 ng/mL lipopolysaccharides (LPS, L5543, Sigma–Aldrich, St. Louis, MO, USA) and 20 ng/mL interferon-γ (IFNγ, I4777, Sigma–Aldrich, St. Louis, MO, USA) in PBS (17-512F, Lonza, Basel, Switzerland) or PBS only (vehicle). Samples were collected 24 h after LPS and IFNγ-mediated induction of neuroinflammation.

For transductions with bicistronic lentiviral particles, BV2 cells were plated at a density of 1 × 10^5^ cells/well. Lentiviral particles were added to each well in 110 µL PBS at MOI 10 and the medium was replaced after 24 h. ZsGreen1 expression was detected 72 h after transduction by fluorescence microscopy. To reduce the cell density before treatments, BV2 cells were transferred to new cell culture plates at a density of 1.5 × 10^5^ cells/well. Then, four hours after plating, cells were treated with LPS and IFNγ for 24 h to induce neuroinflammation, as described above.

Prior to transduction of primary cortical mouse neurons on DIV4, half of the culture medium was collected and replaced with fresh feeding medium. The cells were transduced with lentiviral particles at MOI 10. After 24 h, the medium containing lentiviral particles was replaced with the previously collected medium mixed 1:2 with fresh feeding medium. Neuroinflammation was induced 2 h after addition of BV2 cells on DIV6 with 200 ng/mL LPS and 20 ng/mL IFNγ in PBS or PBS only (vehicle). Samples were collected 48 h later.

### 2.6. RNA Extraction and RT-qPCR Analysis

For RT-qPCR analysis and RNA sequencing, RNA was extracted with High Pure RNA Isolation Kit (11828665001, Roche, Basel, Switzerland). Briefly, cells were scraped in PBS, lysed, and nucleic acids were immobilized by glass fiber fleece filters. Samples were treated with DNase I, and RNA was washed twice before elution. Sample quantitation and purity were determined with a NanoDrop spectrophotometer. For RT-qPCR analysis, RNA was reverse-transcribed using Transcriptor First Strand cDNA Synthesis Kit (04896866001, Roche, Basel, Switzerland) following the manufacturer’s instructions for cDNA synthesis with random hexamer primers. Target specific RT-qPCR primers for mouse *Gapdh* (5′-AACTTTGGCATTGTGGAAGG-3′ and 5′-ACACATTGGGGGTAGGAACA-3′), *β-actin* (5′-GGCTGTATTCCCCTCCATCG-3′ and 5′-CCAGTTGGTAACAATGCCATGT-3′), Il6 (5′-GGGAAATCGTGGAAATGAGA-3′ and 5′-TGAAGGACTCTGGCTTTGTCT-3′), *Tnf* (5′-CGAGTGACAAGCCTGTAG-3′ and 5′-GTGGGTGAGGAGCACGTA-3′), *Il2ra* (5′-CGCTTGAGTCGGCAAAGAAAT-3′ and 5′-CAGAGAGAGATGGTCAATGGC-3′), *Hmox1* (5′-GTCAGGTGTCCAGAGAAGGC-3′, 5′-GCGTGCAAGGGATGATTTCC-3′), *P2ry12* (5′-GCTTGGCAACTCACCTTCAC-3′, 5′-AGGCAGCCTTGAGTGTTCTTC-3′), *Trem2* (5′-TGGAACCGTCACCATCACTC-3′, 5′-TGGTCATCTAGAGGGTCCTCC-3′), *Cst7* (5′-GTGAAGCCAGGATTCCCCAA-3′, 5′-GCCTTTCACCACCTGTACCA-3′), *Bdnf* (5′-TGGCTGACACTTTGAGCAC-3′, 5′-GTTTGGGCATCCAGGTAAT-3′), *Nlgn1* (5′-ACCGTTGGAGCAATTCAACC-3′, 5′-CTATGTGGTATCGGGCTGGT-3′), and human and mouse *MECP2* (5′-TATTTGATCAATCCCCAGGGAAA-3′ and 5′-CTCCCTCTCCCAGTTACCGT-3′) were obtained from TAG Copenhagen (Frederiksberg, Denmark). RT-qPCR was performed using a LightCycler 480 instrument II (05015278001, Roche, Basel, Switzerland) and LightCycler 480 SYBR Green I Master Mix (04887352001, Roche, Basel, Switzerland). The 2^-ΔΔCt^ method was used to calculate the relative fold gene expression of target mRNAs normalized to *Gapdh* or *β-actin*.

### 2.7. Protein Extraction and Western Blot Analysis

Protein lysates for Western blot analysis were prepared by washing the cultured cells twice with PBS and lysing them in T-PER Tissue Protein Extraction Reagent (78510, Thermo Fisher Scientific, Waltham, MA, USA) supplemented with protease and phosphatase inhibitor cocktails (87785 and 78420, Thermo Fisher Scientific, Waltham, MA, USA). To ensure complete cell lysis, the suspension was incubated for 30 min on ice, followed by centrifuging for 10 min at 10,000× *g* for removal of cell debris. Protein concentration was determined using the Pierce BCA Protein Assay Kit (23227, Thermo Fisher Scientific, Waltham, MA, USA) and 10–25 µg total protein samples were incubated for 10 min at 55 °C with NuPAGE LDS Sample Buffer (NP0007, Invitrogen, Carlsbad, CA, USA) before separation on NuPAGE 4–12% BisTris Midi Protein Gels (WG1202BOX, Invitrogen, Carlsbad, CA, USA). SeeBlue Plus2 Pre-stained Protein Standard (LC5925, Invitrogen, Carlsbad, CA, USA) was used to determine the size of proteins. Proteins were transferred onto PVDF membranes (IB24001, Thermo Fisher Scientific, Waltham, MA, USA) with an iBlot 2 Gel Transfer Device (IB21001, Thermo Fisher Scientific, Waltham, MA, USA). The blots were probed at 4 °C overnight with the following antibodies: Phospho-MECP2 Ser80 (1:1000, P21953, Thermo Fisher Scientific, Waltham, MA, USA), Myc Tag (1:1000, Merck Millipore, Burlington, MA, USA), β-actin (1:1000, ab8226, Abcam, Cambridge, UK), HDAC2 (1:1000, ab32117, Abcam, Cambridge, UK), and STAT3 (1:2000, 4904, Cell Signaling Technology, Danvers, MA, USA). After incubation with mouse or rabbit immunoglobulin G (IgG) horseradish peroxidase linked secondary antibody (GENA931 or NA934, GE Healthcare, Chicago, IL, USA), proteins were detected using ECL Select Western Blotting Detection Reagent (RPN2235, GE Healthcare, Chicago, IL, USA) and a ChemiDoc Imaging System (Bio-Rad Laboratories, Hercules, CA, USA). Protein levels were quantified with Image Lab Software 6.0.1 (Bio-Rad Laboratories, Hercules, CA, USA).

### 2.8. Il6, Tnf and Nitrite Assay

For the detection of IL6, TNF, and nitrite, conditioned medium was collected from cultured cells prior to cell collection and centrifuged at 10,000× *g* for 10 min. IL6 and TNF levels were determined using IL6 Mouse ELISA Kit (88-7064-22, Thermo Fisher Scientific, Waltham, MA, USA) and TNF alpha Mouse ELISA Kit (88-7324-22, Thermo Fisher Scientific, Waltham, MA, USA). Nitrite levels were quantitated with the Griess Reagent Kit (G-7921, Invitrogen, Carlsbad, CA, USA). All protein levels were determined according to the manufacturer’s instructions and normalized to total protein concentration of cell lysates from the respective well.

### 2.9. Phagocytosis Assay

For quantification of phagocytosis in vitro, BV2 cells were plated on 96-well cell culture plates (161093, Nunc A/S, Roskilde, Denmark) 7 days after transduction with bicistronic lentiviral particles at a density of 4000 cells/well in 75 µL culture medium. After 16 h, pHrodo Red Zymosan Bioparticles (P35364, Invitrogen, Carlsbad, CA, USA) were added at a final concentration of 5 ng/µL in 25 µL. As a negative control, 2.5 µM phagocytosis inhibitor Cytochalasin D (CytD) was added to two wells per assay. Immediately after addition of pHrodo, fluorescence images were taken every 0.2 h over 1.5 h using a 10× magnification with the IncuCyte S3 Live-Cell Analysis System (Essen BioScience, Ann Arbor, MI, USA) in a humidified incubator at 37 °C in 5% CO_2_. IncuCyte S3 software was used to determine phagocytic activity of transduced BV2 cells by quantifying the area of overlapping pHrodo and ZsGreen1 signal and normalizing it to the ZsGreen1 positive area. As negative controls for image analysis, ZsGreen1-negative BV2 cells and BV2 cells without addition of pHrodo Red were included.

### 2.10. Cytotoxicity Assay

Lactate dehydrogenase (LDH) activity was determined from conditioned medium samples using the Cytotoxicity Detection Kit (11644793001, Roche, Basel, Switzerland) following the manufacturer’s guidelines. Media samples were stored at −80 °C prior to the measurement.

### 2.11. Neuronal Viability Assay

Neuronal viability from mouse primary cortical neuron and BV2 cell co-cultures was assessed by microtubule-associated protein 2 (MAP2) immunostaining as described earlier [31]. Briefly, the cells were fixed and permeabilized and endogenous peroxidase activity was blocked. Cells were incubated with mouse anti-MAP2 primary antibody (1:2000, M9942, Sigma–Aldrich, St. Louis, MO, USA), biotinylated horse anti-mouse secondary antibody (1:500, BA-2000, Vector Laboratories, Burlingame, CA, USA), and ExtrAvidin-Peroxidase (1:500, E2886, Sigma–Aldrich) successively. Color was developed using ABTS Peroxidase Substrate kit (SK-4500, Vector Laboratories, Burlingame, CA, USA) or TMB Peroxidase Substrate Kit (SK-4400, Vector Laboratories, Burlingame, CA, USA) according to the manufacturer’s instructions. Absorbance was measured using a microtiter plate reader (Infinite M200, Tecan) at 405 nm (ABTS) or 650 nm (TMB). Background absorbance was assessed from wells incubated without primary antibody and subtracted from the absorbance value of sample wells.

### 2.12. RNA Sequencing (RNA-seq)

RNA integrity was measured with an Agilent 2100 Bioanalyzer system. Library preparation and RNA sequencing was conducted by Novogene (Cambridge, UK). In brief, mRNA enrichment was performed with oligo(dT) bead pulldown, from where pulldown material was subjected to fragmentation, followed by reverse transcription, second strand synthesis, A-tailing, and sequencing adaptor ligation. The final amplified and size selected library comprised of 250–300 bp insert cDNA and paired-end 150 bp sequencing was executed with an Illumina high-throughput sequencing platform. Sequencing yielded 24–29 million reads per sample.

### 2.13. RNA-seq Data Processing

Examination of the RNA sequencing read quality was performed using FastQC version 0.11.8 [32]. Cutadapt version 2.8 [33] was used for the removal of Illumina sequencing adapter sequences and quality trimming of reads. After trimming, the reads were mapped against the ribosomal reference sequences (build mm10) using Bowtie 2 version 2.2.3 [34]. Reads, which were not successfully mapped, were pseudo-aligned to the mouse reference transcriptome (build mm10) and transcript abundance was quantified using kallisto version 0.46.1 [35]. R package tximport version 1.14 [36] was used to collapse transcript abundance estimates to gene-level counts. Pre-filtering of gene-level counts (mean of gene count > 10) was accomplished using R package DESeq2 version 1.26 [37].

To assess potential batch effects, a principal component analysis (PCA) was performed on the count matrix, whereafter principal components (PC) 1–6 were regressed against known metadata. The resulting R squared values were used to estimate the relation of each PC to the metadata and the need for batch removal. Combat-seq was used to remove a batch effect resulting from RNA extraction days [38].

### 2.14. RNA-seq Analysis

The DESeq2 Wald test was utilized to determine differentially expressed (DE) genes between groups (false discovery rate (FDR) < 0.1) [37]. A correlation matrix for each set of DE genes was generated and clustered (ward.D2) to determine genes showing similar expression alterations between samples and groups. The optimal number of clusters was determined by the Elbow method (silhouette). Gene ontology enrichment analysis (hypergeometric distribution test) was performed to determine overrepresentation of genes in the defined clusters, associated with specific biological processes. Cell type enrichment was determined by one tailed Fisher’s exact test referencing cluster genes with genes determined to be preferentially expressed in neurons or microglia in mouse brain [39].

### 2.15. Statistical Analyses

Apart from RNA-seq data analysis, to test for statistically significant differences between two groups, independent samples t-test or independent samples Mann–Whitney U test was used depending on the distribution of the data. To compare three or more groups, One-way ANOVA, followed by an LSD post-hoc test, was used if data were normally distributed and passed Levene’s test for equality of variances. Otherwise, a Kruskal–Wallis test, followed by Dunn’s multiple comparisons test, was used for the analysis. Statistical significance was set at *p* < 0.05. All statistical analyses were carried out using R [40], SPSS Statistics 25 (IBM, Armonk, NY, USA) and Prism 8 (GraphPad Software, San Diego, CA, USA) software.

## 3. Results

### 3.1. MECP2 Phosphorylation Is Altered at a Functionally Relevant Phosphorylation Site in the Early Stages of AD-Related Neurofibrillary Pathology in Human Brain

**Figure 1:**
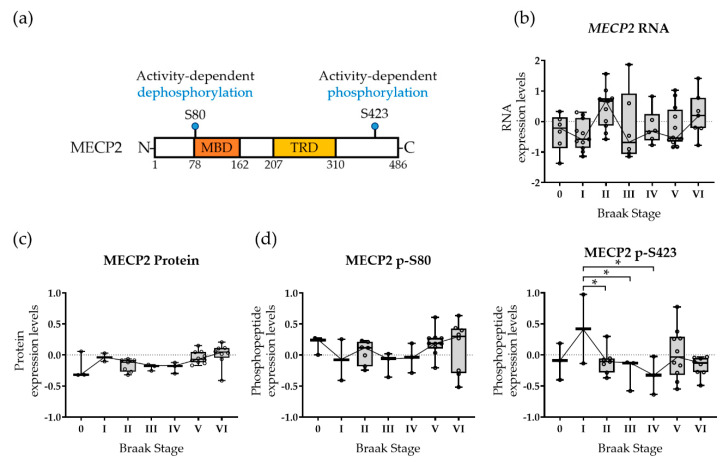
Methyl-CpG-binding protein 2 (MECP2) phosphorylation status is altered in a functionally relevant phosphorylation site with respect to Alzheimer’s disease (AD)-related neurofibrillary pathology in human brain. (**a**) Human MECP2 protein isoform 1 (MECP2A) with its functional domains: the methyl-CpG-binding domain (MBD) and the transcription repression domain (TRD). The positions of two functionally relevant phosphorylation sites, S80 and S423, are indicated in blue. (**b**) Expression of *MECP2* transcript in relation to AD-related neurofibrillary pathology (Braak stage 0–VI) in human brain. Boxplots show median, 25th and 75th percentiles, whiskers show minimum and maximum of *n* = 59 brain samples. Number of samples in each Braak stage 0–VI group: *n* = 6, 11, 11, 6, 7, 12, and 7, respectively. One-way ANOVA, post-hoc LSD. (**c**) Expression of MECP2 protein in relation to AD-related neurofibrillary pathology in human brain. Boxplots show median, 25th and 75th percentiles, whiskers show minimum and maximum of *n* = 36 brain samples. Number of samples in each Braak stage 0–VI group: *n* = 3, 2, 7, 3, 3, 10, and 8, respectively. One-way ANOVA, post-hoc LSD. (**d**) Expression of MECP2 phospho-peptides in relation to AD-related neurofibrillary pathology in human brain. Boxplots show median, 25th and 75th percentiles, whiskers show minimum and maximum of *n* = 36 brain samples. Number of samples in each Braak stage 0–VI group: *n* = 3, 2, 7, 3, 3, 10, and 8, respectively. One-way ANOVA, post-hoc LSD; * *p* < 0.05.

First, we utilized our previous data obtained from a global transcriptomic and phospho-proteomic screening of neuropathologically validated post-mortem temporal cortical samples [27]. The brain samples were originally collected from individuals with a varying degree of AD-related neurofibrillary pathology according to Braak staging (0-VI) [41]. Given the increasing evidence that MECP2 is closely associated with microglial activation and synaptic dysfunction in pathophysiological conditions [8,9,10], we elucidated RNA, protein, and phosphorylation changes of MECP2 in this brain sample set. While the MECP2 RNA (Figure 1b), protein (Figure 1c) and S80 phospho-peptide (Figure 1d) levels remained unchanged, we found a nominal decrease in the functionally relevant MECP2 S423 phosphorylation site (Figure 1a) in the Braak stages II–IV, but not in the Braak stages V or VI (Figure 1d). To further assess the potential links of MECP2 to the pathological changes typical for AD, the levels of soluble Aβ42 measured from temporal cortical samples as well as the levels of Aβ42, p-tau, and total-tau measured from CSF samples were correlated to the RNA, protein, and phosphorylation changes of MECP2 (Figure S1). Consequently, we identified a significant negative correlation between the levels of *MECP2* RNA and Aβ42 in the CSF. Furthermore, as myocyte enhancer factor 2C (MEF2C) has been shown to regulate the expression of *MECP2* [42], we analyzed the expression of *MEF2C* in relation to that of *MECP2*, but did not find a significant correlation between these transcripts (Pearson’s r = −0.12, *p* = 0.35). These findings suggest that PTMs, rather than alterations in the overall expression of MECP2 may associate with pathophysiological changes in the brain in the early stages of AD-related neurofibrillary pathology.

### 3.2. MECP2 Overexpression Increases the Expression of Pro-Inflammatory Cytokines in BV2 Cells upon LPS/IFNγ-Induced Neuroinflammation

**Figure 2:**
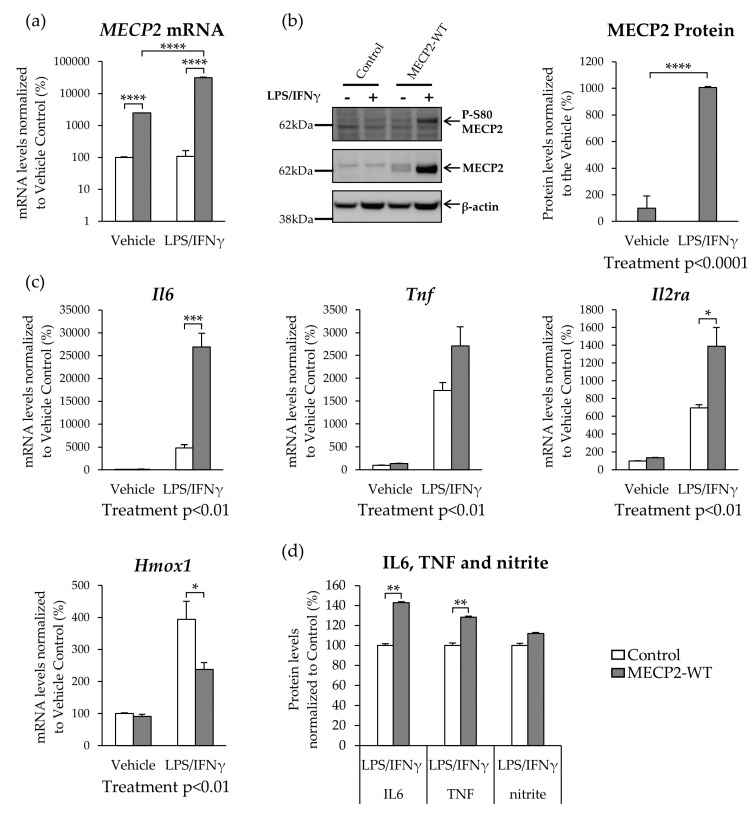
MECP2 overexpression increases the expression of pro-inflammatory cytokines in BV2 cells upon lipopolysaccharide (LPS) and interferon-γ (IFNγ)-induced inflammation. BV2 cells were transduced with lentivirus vectors encoding MECP2-Myc-DDK or tags only (Control) under human cytomegalovirus (CMV) promoter. Inflammation was induced by treatment with LPS (200 ng/mL) and IFNγ (20 ng/mL) for 24 h. (**a**) *Gapdh*-normalized *MECP2* mRNA expression. Data are shown as mean + SEM of *n* = 3. One-way ANOVA, post-hoc LSD; **** *p* < 0.0001. (**b**) A representative Western blot image showing MECP2-Myc-DDK overexpression (anti-Myc tag primary antibody) and phosphorylation at serine 80 (S80) epitope in vehicle and LPS/IFNγ-treated BV2 cells (left). Quantification of *β-actin*-normalized exogenous MECP2 protein levels (right). Data are shown as mean + SEM of *n* = 7 of two independent experiments. Independent samples t-test; **** *p* < 0.0001. (**c**) *Gapdh*-normalized mRNA expression of pro-inflammatory cytokines *Il6*, *Tnf*, and *Il2ra*, and anti-inflammatory *Hmox1*. Data are shown as mean + SEM of *n* = 7 of two independent experiments. Independent samples t-test or independent samples Mann–Whitney U test; * *p* < 0.05, ** *p* < 0.01, *** *p* < 0.001. (**d**) IL6, TNF and nitrite levels in the conditioned medium of BV2 cells. Data are shown as mean + SEM of *n* = 7 of two independent experiments. Independent samples t-test; ** *p* < 0.01.

Since the role of MECP2 in AD pathophysiology is extensively studied in neurons, we first focused on the effects of MECP2 on microglia. Before addressing the phosphorylation sites of MECP2, the effects of wild type MECP2 were first elucidated by transducing BV2 cells with lentiviral vectors encoding MECP2-Myc-DDK (MECP2-WT) or tags only (Control) under the human cytomegalovirus (CMV) promoter. To induce neuroinflammation, BV2 cells were treated with LPS and IFNγ. Under basal conditions, *MECP2* mRNA levels were increased 25-fold in MECP2-WT overexpressing BV2 cells as compared to control cells (Figure 2a). LPS/IFNγ-treatment led to 10-fold higher *MECP2* transcript levels in MECP2-WT overexpressing cells as compared to vehicle-treated cells. In contrast, endogenous *MECP2* mRNA levels in control cells did not increase in response to induction of inflammation. A concomitant significant increase was observed in MECP2 protein levels upon the induction of inflammation in MECP2-WT overexpressing cells (Figure 2b). The protein levels of exogenous MECP2 were 10-fold higher upon LPS/IFNγ treatment as compared to vehicle-treated control cells. Moreover, Western blot analysis showed that the exogenous MECP2 protein wasphosphorylated at S80 upon LPS/IFNγ–induced inflammation in BV2 cells overexpressing MECP2-WT.

To investigate the effects of MECP2-WT overexpression on the inflammatory response in BV2 cells after LPS/IFNγ-treatment, we next determined the mRNA and protein levels of certain pro- and anti-inflammatory molecules. Similar to our previous study, [30], LPS/IFNγ treatment increased the the expression and secretion of pro-inflammatory molecules in BV2 cells (Figure 2c,d). The LPS/IFNγ-induced increase in the levels of overexpressed MECP2-WT was associated with an exacerbated inflammatory response when compared to cells transduced with the control vector. This was indicated by a significant increase in the transcript levels of *Il6, Tnf* and *Il2ra,* while at the same time, the mRNA levels of the anti-inflammatory *Hmox1* were decreased (Figure 2c). The protein levels of the proinflammatory cytokines IL6 and TNF were also increased in the culture medium of BV2 cells overexpressing MECP2-WT when compared to control cells upon LPS/IFNγ-treatment (Figure 2d). A similar increasing trend was observed in the levels of nitrite, the primary breakdown product of NO, but this did not reach statistical significance (Figure 2d).

Previously, it has been suggested that microglial toxicity is mediated by the increased expression of the major glutamine transporter, *Snat1,* in MECP2-deficient microglia [9]. Here, overexpression of MECP2-WT in BV2 cells increased the *Snat1* mRNA levels under basal conditions, while the levels of another amino acid transporter, *Snat2,* were unaffected (Figure S2). Taken together, LPS/IFNγ-induced inflammation promotes the expression of the exogenously expressed MECP2-WT driven by the CMV promoter, which leads to an enhanced pro-inflammatory response in BV2 cells.

### 3.3. Overexpression of Phosphorylation-Deficient Variants of MECP2 Increases the Expression of Pro-Inflammatory Cytokines in BV2 Cells upon LPS/IFNγ-Induced Activation

**Figure 3:**
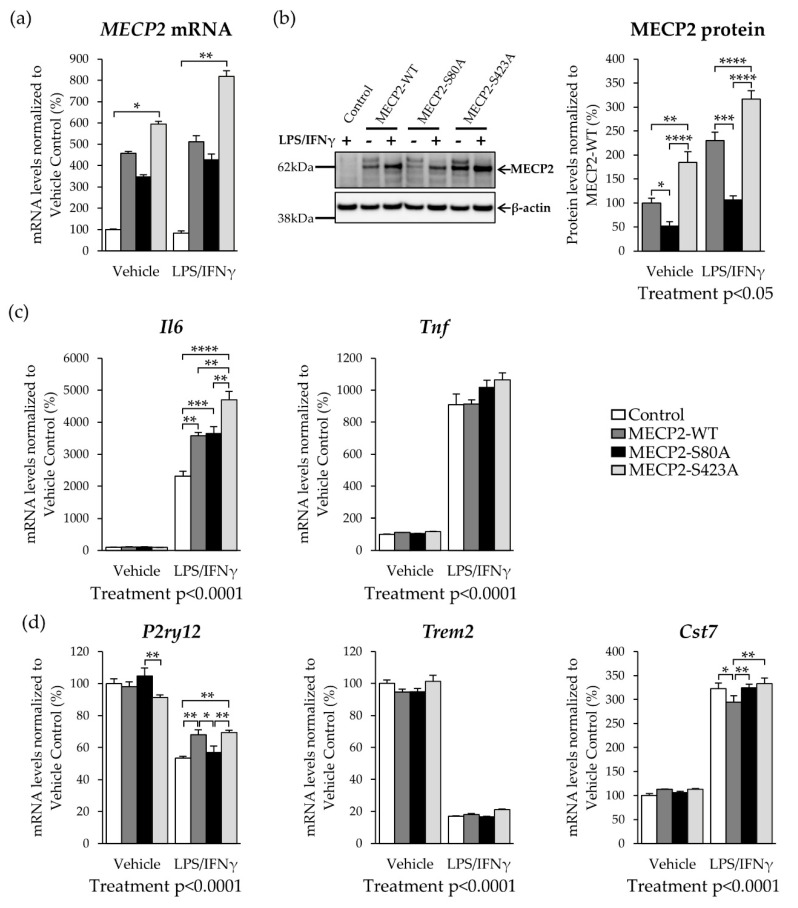
Overexpression of MECP2 phosphorylation-deficient variants increases the expression of pro-inflammatory cytokines in BV2 cells upon LPS/IFNγ-induced inflammation. BV2 cells were transduced with lentivirus vectors encoding MECP2-wild type (WT)-Myc, MECP2-S80A-Myc, MECP-S423A-Myc, or plasmid backbone only (Control). The vector backbone contained the elongation factor-1 alpha (EF1α) promoter and green fluorescent protein (ZsGreen1) coding sequence separated by an internal ribosome entry site (IRES). Inflammation was induced by treatment with LPS (200 ng/mL) and IFNγ (20 ng/mL) for 24 h. (**a**) *β-actin*-normalized *MECP2*-WT, *MECP2*-S423A and *MECP2*-S80A mRNA expression in vehicle and LPS/IFNγ-treated BV2 cells. Data are shown as mean + SEM of *n* = 4. Kruskal–Wallis followed by Dunn’s multiple comparisons test, ** *p* < 0.01, * *p* < 0.05 (**b**) Representative blot image showing MECP2-WT, MECP2-S423A, and MECP2-S80A overexpression in vehicle and LPS/IFNγ-treated BV2 cells (left). Quantification of β-actin normalized exogenous MECP2 protein levels (right). Data are shown as mean + SEM of *n* = 4. Independent samples t-test; **** *p* < 0.0001. (**c**) *β-actin*-normalized mRNA expression of the pro-inflammatory cytokines *Il6* and *Tnf* upon LPS/IFNγ-induced inflammation in BV2 cells expressing MECP2-WT, MECP2-S80A, MECP2-S423A, or Control vector. Data are shown as mean + SEM of *n* = 4. One-way ANOVA, post-hoc LSD. For Treatment effect: Independent-samples t-test or independent samples Mann–Whitney U test was used; **** *p* < 0.0001, *** *p* < 0.001, ** *p* < 0.01. (**d**) Homeostatic (*P2ry12*) and disease-associated microglia (DAM; *Trem2*/*Cst7*) RNA signature upon LPS/IFNγ-induced inflammation in BV2 cells. Data are shown as mean + SEM of *n* = 4. One-way ANOVA, post-hoc LSD. For Treatment effect: Independent-samples t-test or independent samples Mann–Whitney U test was used; ** *p* < 0.01, * *p* < 0.05.

To rule out the possibility that increased MECP2 RNA and protein levels detected upon LPS/INFγ-treatment were induced by the CMV promoter, we replicated our key findings by expressing MECP2-WT under the elongation factor-1 alpha (EF1α) promoter. This led to 4.5-fold higher *MECP2* mRNA levels in transduced BV2 cells when compared to cells transduced with the control vector (Figure 3a). A trend towards increased mRNA levels of *MECP2* upon LPS/IFNγ-treatment was also observed under the EF1α promoter, but this did not reach statistical significance. However, the exogenous protein levels of MECP2-WT were increased by two-fold upon LPS/IFNγ treatment as compared to vehicle-treated control cells (Figure 3b). These results suggest that the EF1α promoter is less responsive to LPS/IFNγ treatment than the CMV promoter and, thus, suitable to reach a moderate overexpression of MECP2-WT in BV2 cells. In addition to the expression of MECP2-WT, we included two phosphorylation-deficient variants of MECP2 in these experiments. To this end, phosphorylation of MECP2 at the S80 and S423 sites was blocked by mutagenizing these serine residues to alanine, yielding the MECP2-S80A and MECP2-S423A variants, respectively. A similar increase to MECP2-WT in the exogenous RNA and protein levels of the MECP2 phosphorylation-deficient variants upon LPS/IFNγ-treatment was detected (Figure 3a).

In line with the results obtained in the BV2 cells overexpressing MECP-WT under the CMV promoter, *Il6* mRNA levels were significantly increased upon LPS/IFNγ-treatment in BV2 cells overexpressing the MECP2-WT under the EF1α promoter. *Il6* transcript levels in MECP2-S80A overexpressing cells were similarly increased to those in the MECP2-WT overexpressing cells. Interestingly, blocking the S423 phosphorylation further increased the expression of *Il6* as compared to MECP2-WT or MECP2-S80A cells (Figure 3c). A previous study by Periyasamy et al. [6] suggested that the increased expression of MECP2 can lead to enhanced expression of *Il6* through the MECP2-STAT3 signaling axis in the mouse primary microglia. To test whether the observed increase in the *Il6* mRNA levels was facilitated through this pathway, we determined the STAT3 protein levels in BV2 cells overexpressing MECP2-WT and the phosphorylation-deficient MECP2 variants in comparison to control cells under basal and inflammatory conditions (Figure S3). STAT3 levels were increased upon LPS/IFNγ-treatment in all the cells as compared to vehicle treatment, and the expression of the MECP2 variants did not lead to significant changes in STAT3 protein levels. Several studies have shown that MECP2 can interact with HDAC and SIN3A to function as a transcriptional repressor [15,43,44,45]. Therefore, we also determined the protein levels of HDAC2, but these did not differ between the control and MECP2-WT or MECP2-S80A cells (Figure S3). MECP2-S423A overexpressing cells showed slightly increased HDAC2 levels, but this difference did not reach statistical significance.

Next, we further characterized the response of the BV2 cells expressing the different MECP2 phosphorylation-deficient variants to LPS/IFNγ-treatment by analyzing the expression of homeostatic and disease-associated microglial (DAM) genes [46,47] (Figure 3d). LPS/IFNγ treatment decreased the expression of the homeostatic marker *P2ry12* by 20–40%. Expression of one of the core DAM genes, *Trem2,* was decreased by 80%, while the mRNA levels of another DAM-related gene, *Cst7,* were increased ~3-fold upon LPS/IFNγ treatment. In line with the increased cytokine levels, treatment with LPS and IFNγ led to a proinflammatory activation rather than DAM activation in BV2 cells. Under inflammatory conditions, BV2 cells expressing MECP2-WT or MECP2-S423A showed a 10% higher expression of *P2ry12* as compared to control or MECP2-S80A expressing cells. While the expression of *Trem2* did not significantly differ between the groups, the expression of *Cst7* was decreased in MECP2-WT expressing cells as compared to the other groups upon LPS/IFNγ-treatment. In summary, a moderate overexpression of MECP2 enhances the pro-inflammatory response of BV2 cells to LPS/IFNγ treatment independently of S80 and S423 phosphorylation.

### 3.4. Overexpression of MECP2 Decreases the Phagocytosis of Zymosan Bioparticles in BV2 Cells Independently of MECP2 Phosphorylation at Serine 80 or Serine 423

**Figure 4:**
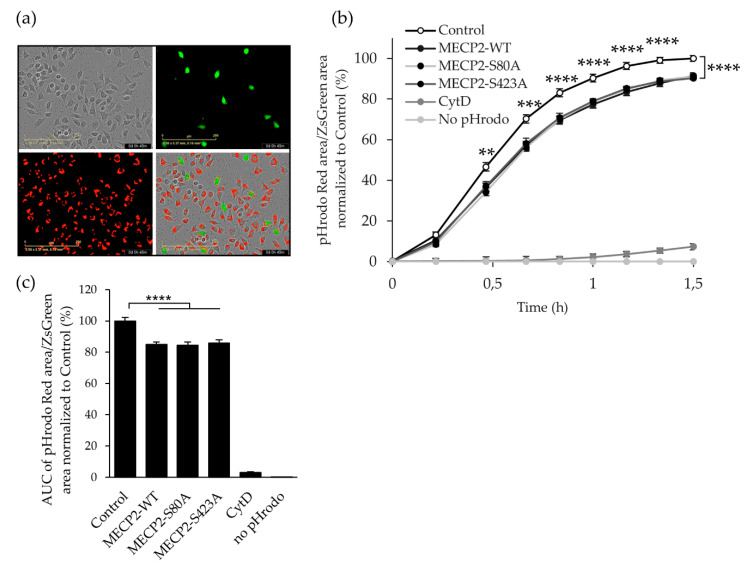
Overexpression of MECP2 decreases zymosan particle phagocytosis in BV2 cells independently of MECP2 phosphorylation at serine 80 or serine 423. BV2 cells were transduced with lentivirus vectors encoding MECP2-WT-Myc, MECP2-S80A-Myc, MECP-S423A-Myc, or plasmid backbone only (Control) seven days prior to phagocytosis assay. The vector backbone contained the elongation factor-1 alpha (EF1α) promoter and green fluorescent protein (ZsGreen1) coding sequence separated by an internal ribosome entry site (IRES). Cells were incubated with pHrodo-labeled zymosan particles (5 ng/µL) and fluorescence emission was measured using the IncuCyte S3 imaging platform every 0.2 h for 1.5 h at 37 °C. (**a**) Representative images showing phase-contrast (BV2 cells), green fluorescence (ZsGreen1) and red fluorescence (pHrodo) signal and overlay 0.75 h after addition of pHrodo-labeled zymosan particles. (**b**) Red pHrodo signal was quantified in ZsGreen-positive areas. To control the assay, 2.5 µM cytochalasin D (CytD) was used as an inhibitor of phagocytosis. Data are shown as Mean ± SEM of *n* ≥ 8 of two independent experiments. One-way ANOVA, post-hoc LSD from each timepoint, ** *p* < 0.01, *** *p* < 0.001, **** *p* < 0.0001. (**c**) Overall phagocytosis measure area under the curve (AUC). Data are shown as Mean + SEM of *n* ≥ 8 of two independent experiments. One-way ANOVA, post-hoc LSD, **** *p* < 0.0001.

Next, we analyzed the phagocytic activity of BV2 cells expressing exogenous MECP2-WT and the phosphorylation-deficient variants under basal conditions using IncuCyte-based live cell imaging (Figure 4a). Quantification of the pHrodo-positive area within ZsGreen1-positive BV2 cells (Figure 4b) revealed a significant decrease in the phagocytic activity of BV2 cells overexpressing the MECP2-WT or the phosphorylation-deficient variants as compared to control cells already at 28 min after the start of the assay, and at all the following time points until the end of the assay. Quantification of total phagocytosis (Figure 4c), using area under the curve (AUC), confirmed the decrease in the phagocytic activity of these cells. No difference was observed between the BV2 cells expressing the MECP2-WT or the phosphorylation-deficient variants, suggesting that the decrease in the phagocytic activity is independent of MECP2 S80 and S423 phosphorylation.

### 3.5. Expression of MECP2-S423A in Mouse Primary Cortical Neurons Co-Cultured with BV2 Cells Leads to an Increased Expression of Bdnf and Nlgn1 upon LPS/IFNγ-Induced Activation

**Figure 5:**
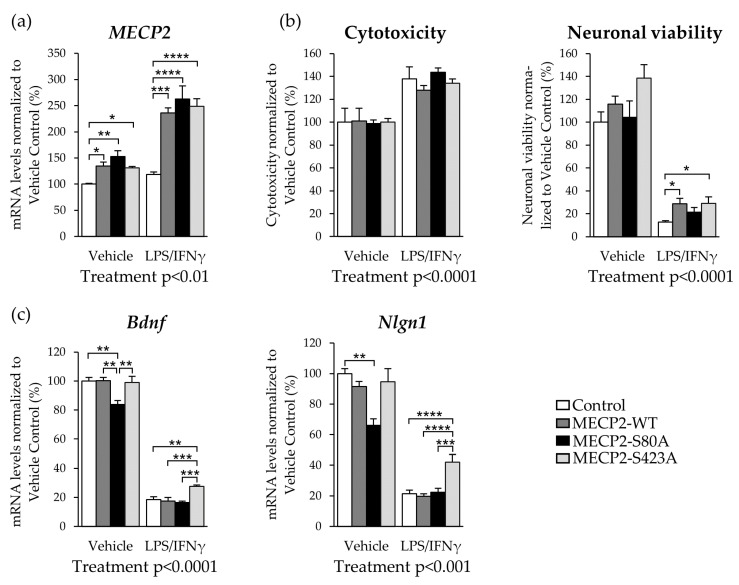
Expression of MECP2-S423A in mouse primary cortical neurons co-cultured with BV2 cells enhances neuronal viability and leads to an increased expression of brain-derived neurotrophic factor (*Bdnf*) and neuroligin 1 (*Nlgn1*) upon LPS/IFNγ-induced activation. Cortical neurons were transduced with lentivirus vectors after four days in vitro (DIV4) encoding MECP2-WT-Myc, MECP2-S80A-Myc, MECP-S423A-Myc, or plasmid backbone only (Control). BV2 cells were added 48 h after the neuronal transduction and inflammation was induced with LPS (200 ng/mL) and IFNγ (20 ng/mL). (**a**) *β-actin*-normalized *MECP2* (mouse and human) mRNA expression in vehicle and LPS/IFNγ-treated neuron-BV2 co-cultures. No treatment effect in comparison to Control group was detected. (**b**) Cytotoxicity (left) and neuronal viability (right) were assessed 48 h after LPS/IFNγ treatment. Cytotoxicity was determined by measuring lactate dehydrogenate (LDH) levels in the conditioned medium. Neuronal viability was assessed by MAP2 immunostaining assay. (**c**) *β-actin*-normalized mRNA expression of *Bdnf* and *Nlgn1* upon LPS/IFNγ-induced inflammation in neuron-BV2 co-cultures. Data are shown as Mean + SEM of *n* = 6–8 of two independent experiments, *n* = 3 for cytotoxicity assay. One-way ANOVA, post-hoc LSD or Kruskal–Wallis and Dunn’s multiple comparisons test. For Treatment effect: Independent-samples t-test or independent samples Mann–Whitney U test was used; **** *p* < 0.0001, *** *p* < 0.001, ** *p* < 0.01, * *p* < 0.05.

As MECP2 phosphorylation at the S80 and S423 sites might not influence microglia alone, we next aimed to investigate the impact of MECP2 phosphorylation in neuron BV2 microglia co-cultures. To determine the effects of changes in the phosphorylation of MECP2 in neuron-BV2 microglia co-cultures, mouse primary cortical neurons were first transduced with MECP2-WT or the phosphorylation-deficient variants, followed by addition of the BV2 cells and induction of neuroinflammation by LPS/IFNγ treatment for 48 h. Under basal conditions, the transduction of the cortical neurons increased the mRNA levels of *MECP2* by 30–50% (Figure 5a). In line with the BV2 cell monoculture data, LPS/IFNγ treatment led to an ~2-fold increase in the exogenous mRNA levels of *MECP2* in neurons as compared to vehicle treatment.

To address the health of the neurons expressing MECP2-WT or the phosphorylation-deficient variants under the LPS/IFNγ-induced pro-inflammatory condition, cytotoxicity, and neuronal viability was next measured. As expected, cytotoxicity increased upon the induction of neuroinflammation (Figure 5b). Accordingly, neuronal viability was decreased upon the LPS/IFNγ treatment in the neuron-BV2 cell co-cultures. Interestingly however, overexpression of MECP2-WT, as well as the MECP2-S423A variant, led to increased neuronal viability by 50% upon induction of neuroinflammation as compared to the control group. Concomitantly, the cytotoxicity assay showed a decreasing trend in the co-cultures of BV2 cells and the MECP2-WT or MECP2-S423A expressing neurons when compared to the control co-cultures.

It has been previously shown that MECP2 can regulate the expression of *Bdnf* and neuroligin 1 (*Nlgn1*), which both play important roles in synaptic plasticity [10,16]. Here, MECP2-WT or MECP2-S423A did not affect the expression of *Bdnf* or *Nlgn1* in the vehicle-treated co-cultures, but both the *Bdnf* and *Nlgn1* mRNA levels were significantly decreased in MECP2-S80A expressing neurons co-cultured with BV2 cells (Figure 5c). LPS/IFNγ treatment decreased the *Bdnf* transcript levels by 80% in all the groups except in MECP2-S423A expressing neurons co-cultured with BV2 cells. In these cells, *Bdnf* expression was only decreased by 70%, suggesting that the phosphorylation of MECP2 at S423 plays a role in the regulation of *Bdnf* expression. A similar expression pattern was observed for *Nlgn1*. The *Nlgn1* mRNA levels were ~2-fold higher in the MECP2-S423A expressing neurons co-cultured with BV2 cells as compared to the other groups upon induction of neuroinflammation. In summary, these findings suggest that phosphorylation of MECP2 at S80 in neurons regulates the gene expression of *Bdnf* and *Nlgn1* in basal conditions, while the phosphorylation of S423 may play a role under inflammatory stress.

### 3.6. Abrogation of MECP2 Phosphorylation at S423 Changes Global Transcription in Primary Cortical Neurons Co-Cultured with BV2 Cells upon LPS/IFNγ-Induced Neuroinflammation

**Figure 6:**
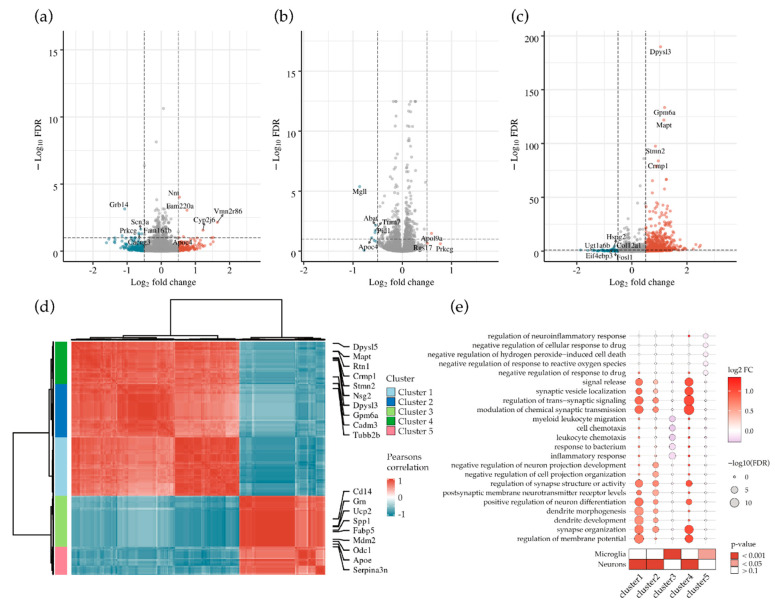
Abrogation of MECP2 phosphorylation at S423 changes global transcription in primary cortical neurons co-cultured with BV2 cells upon LPS/IFNγ-induced neuroinflammation. Volcano plot representation of differentially expressed genes for (**a**) MECP2-WT compared to Control (ZsGreen1), (**b**) MECP2-S80A compared to MECP2-WT and (**c**) MECP2-S423A compared to MECP2-WT expressing primary cortical neurons co-cultured with BV2 cells upon LPS/IFNγ-induced inflammation. Significantly (FDR-adjusted *p*-value < 0.1 y-axis, dotted line) down- (blue, log2 fold-change < 0.5) and upregulated (red, log2 fold-change > 0.5) genes and their log2-transformed fold-changes (x-axis) from *n* = 4 samples. (**d**) Clustered correlation matrix (Pearson correlation) for differentially expressed genes in MECP2-S423A compared to MECP2-WT. Highlighted are the top up- and down-regulated genes determined in differential expression analysis. (**e**) A dot plot to represent the top five enriched gene ontology (GO, biological processes) terms (y-axis) for each cluster (x-axis). The size of the symbol represents the FDR-adjusted *p*-value, the color represents the log2-fold change. Below, a heatmap representing neuron or microglia marker enrichment for each cluster. Color represents the FDR-adjusted *p*-value.

To characterize the expressional changes due to the blocking of MECP2 S423 phosphorylation in neuron-BV2 co-cultures upon induction of neuroinflammation in more detail, a subset of the samples used for *Bdnf* and *Nlgn1* (Figure 5) qPCR analyses was subjected to bulk RNA sequencing (RNA-seq). RNA-seq was performed in primary cortical mouse neurons transduced with MECP2-WT, MECP2-S80A, MECP2-S423A, or ZsGreen1 (Control), co-cultured with BV2 cells and treated with LPS/IFNγ for 48 h. The analysis of differentially expressed (DE) genes showed that the overexpression of MECP2-WT in neurons (Figure 6a) did not have major effects on global gene expression as compared to control co-cultures (202 DE genes, FDR < 0.1). The expression of MECP2-S80A (Figure 6b) did not lead to significant gene expression changes either as compared to MECP2-WT expression (370 DE genes, FDR < 0.1). In contrast, the overexpression of MECP2-S423A (Figure 6c) resulted in major changes in the global gene expression as compared to MECP2-WT (1181 DE genes, FDR < 0.1). MECP2-S423A expression led to an upregulation in the expression of 782 genes. Among the most significantly upregulated genes were Dihydropyrimidinase Like 3 and 5 (*Dpysl3/5*, FDR = 1.2E-190/3.2E-67, log2 fold-change = 1.04/1.24), and their paralog gene Collapsin Response Mediator Protein 1 (*Crmp1*, FDR = 1.37E-88, log2 fold-change = 0.95), which play a role in neuronal differentiation and axon guidance. Interestingly, microtubule associated protein tau (*Mapt*) also showed a prominently increased expression in MECP2-S423A expressing neuron-BV2 co-cultures (FDR = 1.7E-122, log2 fold-change = 1.16). To further investigate the potential relationships between the differentially expressed genes in MECP2-S423A vs. MECP2-WT co-cultures, we generated a correlation matrix and clustered genes with similar expressional alterations between samples and groups (Figure 6d). This resulted in five distinct gene clusters, two of which represented downregulated genes (clusters 3 and 5) and three, which represented upregulated genes (clusters 1, 2, and 6). Gene Ontology (GO, biological processes) cell type enrichment analysis (Figure 6e) showed that genes within the three overexpression clusters are mainly specific for neurons. The upregulated neuronal genes were overrepresented in processes, such as synaptic transmission, synapse organization, and dendrite development. On the other hand, the two clusters representing downregulated genes mainly consisted of microglia-specific genes associated with processes such as cell chemotaxis and inflammatory response. Bulk RNA sequencing data for *Bdnf* and *Nlgn1* expressional changes between the different groups correlated with the results obtained by RT-qPCR analysis (Figure 5c). Taken together, the RNA sequencing data revealed that abrogating the MECP2 S423 phosphorylation in mouse primary cortical neurons results in a profound increase in the expression of target genes relevant for the maintenance and protection of neurons and synapses. Concomitantly, a repression in the expression of genes related to inflammation was detected, potentially reflected by the observed positive impact on neuronal functions.

## 4. Discussion

MECP2 is a key regulator of synaptic plasticity-associated genes and altered function of MECP2 has been shown to mediate synaptic and neuronal dysfunction in different pathophysiological conditions [5,7,8,9]. Furthermore, a role for MECP2 in modulating certain functions of microglia has recently been reported [6,18,20]. Importantly, it has been shown in rodents that Aβ fibril-induced neuroinflammation reduced MECP2-mediated *Nlgn1* expression, which consequently underlay β-amyloid-induced memory deficiency. Thus, these previous findings reinforce the importance of assessing the potential effects and the underlying molecular mechanisms of MECP2 in the cellular processes relevant for AD pathogenesis. Since synaptic dysfunction is the best correlate of cognitive decline in AD and microglia-driven neuroinflammation is one of the first hallmarks of AD pathogenesis, we have here analyzed the expression, protein levels, and phosphorylation changes of MECP2 in relation to AD-related neurofibrillary pathology in human brain. The effects of the wild type and phosphorylation-deficient variants of MECP2 on microglial and neuronal functions were also assessed by utilizing in vitro models, which recapitulate certain fundamental cellular aspects of AD pathology, such as microglial activation via the NF-kB signaling pathway and synaptic dysfunction in neurons [25,26].

First, we showed that the S423 phosphorylation of MECP2 was nominally decreased with respect to AD-related neurofibrillary pathology in human post-mortem temporal cortex, while MECP2 RNA and protein levels were not altered. Since the function of MECP2 as a transcriptional regulator is tightly controlled by PTMs, including phosphorylation at the S423 site, our findings suggest that the activity rather than the levels of MECP2 shows alterations in the early stages of AD-associated neurofibrillary pathology. Importantly, mouse MECP2 S421 phosphorylation has been shown to lead to changes in the MECP2 transcriptional repressor complex, which facilitate the expression of BDNF, and, thus, promote synaptic function and improve learning and memory [15,16]. Collectively, the previous studies and the current findings suggest that the reduced phosphorylation of MECP2 at S423 may contribute to or reflect synaptic dysfunction and cognitive decline in AD. Based on these findings, however, it cannot be concluded if the observed phosphorylation changes in MECP2 are cell-type specific. Thus, single cell-based analyses would be required to find out whether the MECP2 phosphorylation changes affect the functions of neurons and microglia in AD-related cellular processes. In human brain tissue, a significant increase in the microglial activation is observed in relation to advanced AD pathology, which strongly correlates with the occurrence of dystrophic neurites [48]. In the late stages of AD, dystrophic microglia are abundant in the brain tissue, emphasizing the importance of specifically assessing the MECP2-related effects also in this type of microglia. Importantly, bidirectional neuron-microglia communication through the release of soluble factors, such as fractalkine, chemokines, and complement factors, is crucial for synaptic functions [49], and disturbances in neuron-microglia communication have been implicated to play a central role in AD progression [50]. Accordingly, our RNA-seq results from the neuron-microglia co-cultures highlight the importance of interaction between neurons and microglia, as expression of MECP2-S423A in the neurons led to downregulation of certain microglia-specific genes that are involved in the inflammatory response and cell chemotaxis. To further delineate the role of MECP2 phosphorylation changes in neuron-microglia interaction, specific models focusing on the key immune pathways involved in AD pathogenesis, such as the TREM2 signaling and complement cascade [51], are needed.

In contrast to our results obtained from human post-mortem brain, elevated MECP2 protein levels have been reported in the hippocampal tissue of AD patients compared to controls [12]. A large-scale proteomic analysis of AD dorsolateral prefrontal cortex tissue found only a modest increase in MECP2 protein levels at the late stages of the disease [52]. The difference between these findings may be explained by the analyses of different brain areas or by the fact that the first-mentioned study was based on a smaller sample size, comprising of only four AD and four control brain samples. Another study, utilizing immunohistochemical analysis, found increased levels of MECP2 phosphorylated at S80 in the temporal cortical samples from human AD patients as compared to non-AD controls [11]. In contrast, we did not find changes in S80 phosphorylation with respect to AD-associated neurofibrillary pathology. However, it should be noted that there was an inverse correlation of the phosphorylation profile at the S80 site as compared to the S423 with respect to increased neurofibrillary pathology, suggesting a potential link between these two phosphorylation sites in terms of PTMs upon AD pathogenesis. Finally, elucidation of relevant AD-associated metadata available from the post-mortem tissue cohort [27] in relation to RNA, protein, and phosphorylation changes of MECP2, revealed a nominally significant negative correlation between the levels of Aβ42 in the CSF and the *MECP2* RNA levels in the temporal cortex. However, a similar correlation was not observed between the CSF Aβ42 levels and the MECP2 protein levels or the phosphorylation changes in MECP2, emphasizing that further studies are still needed in different cell types to delineate the role of MECP2 in the key pathophysiological processes in AD.

MECP2 has been shown to regulate different functions of microglia [6,18,20], but the role of MECP2 PTMs has not previously been characterized in this context. Here, overexpression of MECP2-WT increased the expression and secretion of pro-inflammatory cytokines in BV2 cells upon LPS/IFNγ-induced activation, suggesting that MECP2 may participate in the regulation of the inflammatory response in microglia. A particularly strong increase was observed in the *Il6* transcript levels. In this context, BV2 cells transduced with a lentivirus vector encoding MECP2 under the CMV promoter displayed a substantial increase in the mRNA and protein levels of MECP2 upon treatment with LPS and IFNγ. Since the CMV promoter has previously been shown to be responsive to LPS and IFNγ [53,54,55,56], we replicated the main experiments after exchanging the CMV promoter to the EF1α promoter. Under the EF1α promoter, MECP2 levels increased in a similar manner to the cells transduced with MECP2 under the CMV promoter, but the increase was less robust. Thus, the subsequent experiments were performed by using lentivector constructs under the EF1α promoter. Interestingly, blocking of the S423 phosphorylation further increased the *Il6* transcript levels as compared to the cells expressing MECP2-WT. Increased IL6 levels have been observed in the plasma and saliva from MECP2-deficient individuals compared to healthy subjects [57,58]. Together with our findings, this suggests that a tight regulation of MECP2 levels is important for IL6 expression. Previously, IL6 expression has been shown to be mediated by the MECP2-STAT3 signaling pathway in microglia [6]. However, the increase detected in the present study was not likely facilitated through the MECP2-STAT3 signaling pathway, since the STAT3 protein levels were not altered upon the overexpression of MECP2. MECP2 can also form a complex with HDAC and SIN3A, and it has been shown that knockdown of *Hdac2* can suppress *Il6* and *Tnf* expression in BV2 cells upon treatment with LPS [59]. Here, HDAC2 protein levels did not alter upon MECP2 overexpression, suggesting that HDAC2 did not facilitate the observed increase in the expression of *Il6* and *Tnf*. In pancreatic adenocarcinoma cell lines, Dandrea et al. found a possible MECP2 binding site in the *IL6* upstream region and chromatin immunoprecipitation experiments in synovial fibroblasts have shown binding of MECP2 to the *IL6* promoter [60,61]. These findings raise the possibility that MECP2 may directly influence the expression of *Il6*. Together, our findings suggest that MECP2 regulates the pro-inflammatory response in microglia, which is at least partially dependent on S423 phosphorylation.

To address microglia functions beyond the inflammatory responses, we also analyzed the expression of some key homeostatic (*P2ry12*) and DAM markers (*Trem2* and *Cst7*) [46,47], as well as the phagocytic capacity of BV2 cells overexpressing WT, S80A and S423A variants of MECP2. In MECP2-WT and MECP2-S423A expressing BV2 cells, the expression of *P2ry12*, a microglia chemotactic receptor, was less reduced as compared to control or MECP2-S80A cells upon LPS/IFNγ-induced inflammation. This observation raises the possibility that the MECP2 overexpressing microglia show an enhanced capacity to respond to chemotactic stimuli. Importantly, chemotaxis-driven microglial motility has been shown to be impaired in mouse models of AD [62]. Interestingly, BV2 microglial cells expressing the MECP2-S80A or S423A variants showed a significantly increased expression of *Cst7* as compared to MECP2-WT-expressing BV2 cells upon the induction of inflammation, suggesting that BV2 cells expressing the phosphorylation-deficient variants show a more prominent microglial activation. Conversely, the expression of *Trem2* showed an expected decrease due to LPS-induced inflammation [63], but there were no statistically significant differences between the control BV2 cells and the BV2 cells expressing MECP2-WT or the phosphorylation-deficient variants in terms of *Trem2* expression. Finally, we observed a reduction in the phagocytic activity in the BV2 cells overexpressing the MECP2-WT or the phosphorylation-deficient variants when compared to control cells. In line with this, a significant increase in the phagocytic activity has been reported in the retinogeniculate system of aged *Mecp2*-null mice in vivo [64]. In contrast, *Mecp2*-null primary microglia showed decreased phagocytosis of apoptotic targets in vitro [21]. In the latter study, microglia were incubated with labeled target cells for two or five hours. Since phagocytosis is a dynamic process, phagocytic activity might differ over time. Additionally, it has been shown that phagocytic uptake also depends on the shape and the size of the target [65]. Despite the differences in these findings, they show that a tight regulation of MECP2 levels in microglia is crucial for the regulation of phagocytic activity. In the context of AD, it has been hypothesized that both reduced and elevated phagocytic activity of microglia may contribute to disease progression [66,67]. While a certain level of phagocytic activity is indispensable for the clearance of apoptotic cells and aggregated proteins, excessive phagocytosis of live neurons and synapses can be detrimental [66,67]. In summary, MECP2 shows both PTM-dependent and independent effects on BV2 microglial activation and phagocytosis upon LPS-induced inflammation. In this context, however, it should be noted that although LPS has been shown to induce microglia-mediated synaptic failure similar to Aβ [10,24], further studies in physiologically more relevant AD models are still needed to recapitulate PTM-dependent and independent effects of MECP2 in chronic neuroinflammation.

MECP2 also has a central role in the regulation of neuronal and synaptic functions [5]. BDNF and NLGN1 are proteins that are involved in synaptic plasticity and exert neuroprotective functions [10,16]. MECP2 has been shown to regulate the expression of both *BDNF* and *NLGN1* [68,69]. However, the role of MECP2 in the regulation of *BDNF* expression is controversial. MECP2 has been proposed to act as a repressor of *Bdnf* gene expression in primary mouse and rat cortical neurons in vitro [68,70]. Later, a functional interaction between MECP2 and *Bdnf* was confirmed in vivo using *Mecp2*-null mice, but counterintuitively to the in vitro findings, the mice showed a downregulation in the RNA and protein levels of BDNF [71]. Furthermore, in MECP2 transgenic (MECP2-Tg) mice, the expression of *Bdnf* was upregulated as compared to WT littermates [45]. We assessed the role of MECP2 and its phosphorylation-deficient variants in mouse cortical neurons co-cultured with BV2 cells under LPS/IFNγ-induced neuroinflammation and focused first on the expression of *Bdnf* and *Nlgn1*. MECP2-WT overexpression in the primary neurons did not lead to changes in *Bdnf* mRNA levels as compared to control cells under basal or inflammatory stress conditions. However, blocking of MECP2-S423 phosphorylation resulted in increased *Bdnf* mRNA levels, indicating that this phosphorylation site is essential for the MECP2-mediated modulation of *Bdnf* expression. This increase was only detectable in LPS/IFNγ-treated co-cultures and could either be caused by the inflammatory stress or by the stronger MECP2 overexpression upon LPS/IFNγ treatment. The expression of *Nlgn1* showed similar alterations upon MECP2 overexpression and LPS/IFNγ treatment to *Bdnf*. A recent study suggested that Aβ oligomers can reduce the levels of NLGN1 and that NLGN1 levels were decreased in the hippocampus of AD patients [72]. Our finding that MECP2-S423A increases the expression of *Bdnf* and *Nlgn1* in vitro suggests that MECP2 dephosphorylation at S423 in AD brains may be an underlying mechanism mediating neuroprotection. Supporting these findings, we found that neuronal viability was increased upon induction of neuroinflammation in cortical neurons expressing the MECP2-S423A variant as well as MECP2-WT. Although not statistically significant, a trend towards concomitantly reduced cytotoxicity was also observed in those two groups upon the LPS/IFNγ treatment. Thus, our results suggest that MECP2 S423 is an essential phosphorylation site mediating the regulation of *Bdnf* and *Nlgn1* expression upon neuroinflammation.

Finally, the bulk RNA sequencing of the neuron-BV2 co-culture samples showed that the overexpression of MECP2-WT or MECP2-S80A did not have major effects on global gene expression under the inflammatory stress. However, the overexpression of MECP2-S423A led to upregulated gene expression of a large number of genes. In vitro binding assays have previously shown that mouse MECP2-S421A binds methylated DNA with a similar affinity to WT MECP2 unphosphorylated at S421, and that S421-phosphorylated MECP2 does not bind methylated DNA [73]. In line with our results, neurons from MECP2 S421A/S424A knock-in mice showed an increase in *Bdnf* expression [17,74], and an increased promoter binding by MECP2-S421A/S424A was found as compared to wild type MECP2 [74]. Thus, the observed changes in the global gene expression in our study suggest that MECP2-S423A encompasses reduced capacity to repress transcription, leading to an increased expression of specific target genes, possibly via altered binding to promoter regions. Importantly, several of the these differentially expressed genes upon MECP2-S423A expression play central roles in the maintenance and protection of neurons and synapses, and some of these genes have previously been shown to associate with MECP2. The expression of *Dpysl3* and *Dpysl5* has been shown to be upregulated in MECP2-Tg mice and downregulated in Rett syndrome, a disorder caused by mutations in the MECP2 gene [45]. Additionally, *Crmp1* is upregulated in MECP2-Tg mice and MECP2 has been reported to associate with the promoter regions of *CRMP1* in SH-SY5Y human neuroblastoma cells [45,75]. Expression of *Stmn2* has been shown to be reduced in the cerebellum of MECP2-deficient mice, as well as in the fibroblasts from Rett syndrome patients [76,77]. Interestingly, human STMN2 protein levels were reduced in AD brains and negatively correlated with the numbers of neurofibrillary tangles [78]. STMN2 has also been shown to interact with amyloid precursor protein (APP) and may play a role in AD pathology [79]. A key AD-associated gene that was also upregulated upon blocking of MECP2-S423 phosphorylation is *Mapt,* which encodes for tau protein. In MECP2-Tg mice, elevated tau levels have been observed in the hippocampus and cortex [80]. In N2a mouse neuroblastoma cells expressing human tau, the knockdown of *Mecp2* led to a decrease in the levels of total and phosphorylated tau and it was suggested that MECP2 is a potential regulator of tau pathology [11]. Our results now show a connection between MECP2 and *Mapt* expression in primary neurons upon neuroinflammation, where MECP2 phosphorylation at S423 may be an important regulatory PTM. RNA sequencing also revealed downregulation of some microglia-specific genes, particularly some involved in the inflammatory response, in neuron-BV2 co-cultures expressing MECP2-S423A as compared to MECP2-WT-expressing cultures. Since MECP2-S423A was only expressed in the neurons, MECP2-S423A-expressing neurons may influence the response of BV2 cells to LPS/IFNγ. Moreover, it has been shown that primary mouse cortical neurons can react to IFNγ even in the absence of glial cells through a neuron-specific IFNγ receptor [81]. Therefore, the expression of MECP2-S423A might alter the response of neurons to IFNγ, which could lead to changed composition of secreted factors dampening the microglial response. Collectively, the results emphasize that the MECP2 S423 phosphorylation site plays a central role in the activation of neuronal gene expression under neuroinflammation-related stress. The previously mentioned mouse study [74] supports our finding that blocking of MECP2 S423 phosphorylation may exert protective effects on synapses and neurons as MECP2 S421A/S424A knock-in mice showed an enhancement of synaptogenesis, long-term potentiation, and learning and memory when compared to WT littermates. Further studies will be needed to address the impact of blocked S423 phosphorylation in human MECP2 on different cells as well as on synapses.

In conclusion, we report here that activity-related phosphorylation of MECP2 at S423 was nominally reduced with respect to AD-associated neurofibrillary pathology in human brain. Functional analyses in in vitro cell models suggest that abrogated phosphorylation at S423 associated with enhanced inflammatory response in microglia. On the other hand, the phosphorylation-deficient MECP2-S423A variant enhanced neuronal viability and increased the expression of several genes, which play important roles in the maintenance and protection of neurons and synapses. Thus, the reduced phosphorylation of S423 in the early stages of AD-related neurofibrillary pathology suggests a potential compensatory effect in AD brain undergoing neurodegeneration, which in turn reinforces the idea that PTMs in MECP2 are potential targets for developing specific therapies against AD and other neurodegenerative diseases.

## Data Availability

The RNA-seq data presented in this study are openly available in Gene Expression Omnibus (GEO) repository at https://www.ncbi.nlm.nih.gov/geo/query/acc.cgi?acc=GSE166670 (reference number GSE166670, accessed on 7 April 2021).

## Supplementary Materials

The following are available online at https://www.mdpi.com/article/10.3390/cells10040860/s1, Figure S1: Correlation of human brain MECP2 RNA, protein, S80, and S423 phosphopeptide levels against CSF Aβ42, phosphorylated-tau (p-tau), and total-tau as well as brain soluble Aβ42 levels. Figure S2: Lentivirus-mediated MECP2 overexpression up-regulates expression of glutamine transporter SNAT1 but not SNAT2 under basal condition in BV2 cells. Figure S3: Overexpression of MECP2 WT and its variants does not significantly affect the levels of HDAC2 or STAT3 upon basal or inflammation-induced conditions in BV2 cells.
