# Supplementary material for: MECP2 Increases the Pro-Inflammatory Response of Microglial Cells and Phosphorylation at Serine 423 Regulates Neuronal Gene Expression upon Neuroinflammation
Source: Cells. 2021 Apr 9;10(4):860. doi: 10.3390/cells10040860 (PMC8070522; doi:10.3390/cells10040860)
Supplement: Supplementary file 1 [file cells-10-00860-s001.pdf]

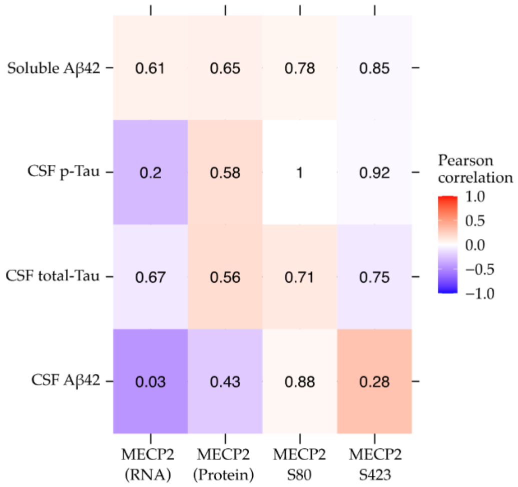

Figure S1. Correlation of human brain MECP2 RNA, protein, S80, and S423 phosphopeptide levels against CSF Aβ42, phosphorylated-tau (p-tau), and total-tau as well as brain soluble Aβ42 levels. Heatmap color represents Pearson's correlation coefficient value, and the value in tile indicates corresponding correlation p-value. RNA: CSF Aβ42: $n = 22$, CSF p-/total-tau: $n = 21$, soluble Aβ42: $n = 55$. Protein/phosphopeptide: CSF Aβ42: $n = 14$, CSF p-/total-tau: $n = 13$, soluble Aβ42: $n = 36$.

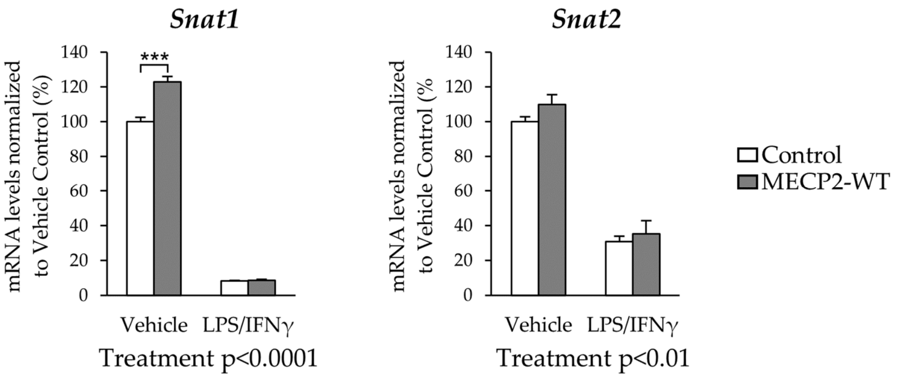

Figure S2. Lentivirus-mediated MECP2 overexpression up-regulates expression of glutamine transporter Snat1 but not Snat2 under basal condition in BV2 cells. *Gapdh*-normalized mRNA expression of glutamine transporters *Snat1* and *Snat2* upon LPS/IFNγ-induced inflammation in BV2 cells transduced with lentiviral vectors encoding MECP2-Myc-DDK or tags only (Control). Data shown as mean + SEM of $n = 6$-7. Independent-samples t-test or independent samples Mann-Whitney U test; ***$p < 0.001$.

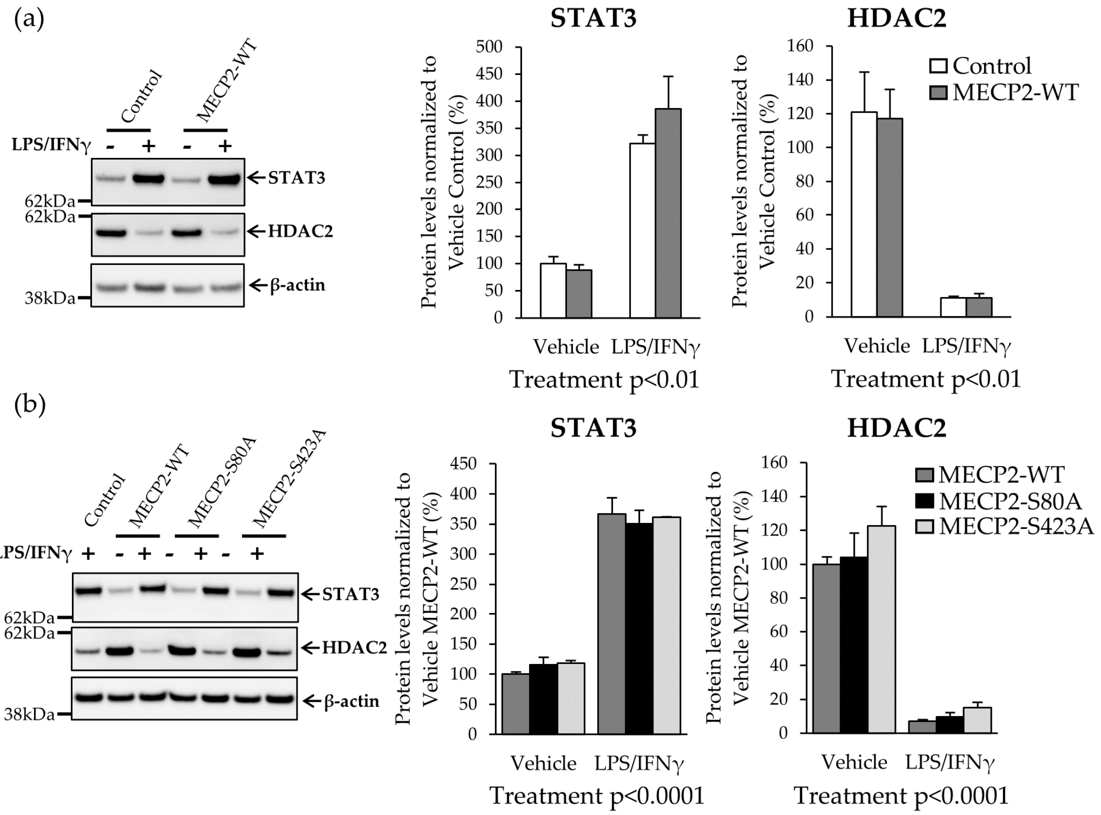

Figure S3. Overexpression of MECP2 WT and its variants does not significantly affect the levels of HDAC2 or STAT3 upon basal or inflammation-induced conditions in BV2 cells. BV2 cells were transduced with lentiviral vectors encoding MECP2-WT-Myc, MECP2-S80A-Myc MECP-S423A-Myc or vector backbone only (Control). Vector backbone contained EF1α (elongation factor-1 alpha) promoter and ZsGreen1 (green fluorescent protein) coding sequence separated by IRES (internal ribosome entry site). Inflammation was induced by treatment with LPS (200 ng/ml) and IFNγ (20 ng/ml) for 24 h. **(a)** Representative blot image showing STAT3 and HDAC2 expression in vehicle and LPS/IFNγ-treated BV2 cells comparing ZsGreen1 expressing cells (Control) to MECP2-WT expressing cells. Graphs showing quantification of β-actin normalized STAT3 and HDAC2 protein levels. Data shown as mean + SEM of n = 3. Independent samples t-test. **(b)** Representative blot image showing STAT3 and HDAC2 expression in vehicle and LPS/IFNγ-treated BV2 cells expressing MECP2-WT, MECP2-S80A or MECP2-S423A. Graphs showing quantification of β-actin normalized STAT3 and HDAC2 protein levels. Data shown as mean + SEM of n = 3. One-way ANOVA, post-hoc LSD. For treatment effect: Independent-samples t-test.
